# Characterization of a highly xylose tolerant β-xylosidase isolated from high temperature horse manure compost

**DOI:** 10.1186/s12896-021-00722-6

**Published:** 2021-10-24

**Authors:** Kanyisa Ndata, Walter Nevondo, Bongi Cekuse, Leonardo Joaquim van Zyl, Marla Trindade

**Affiliations:** 1 Institute for Microbial Biotechnology and Metagenomics, Department of Biotechnology, University of the Western Cape, Bellville, South Africa; 2 Division of Medical Virology, Institute of Infectious Disease and Molecular Medicine, Faculty of Health Sciences, University of Cape Town, Cape Town, South Africa

**Keywords:** Metagenomics, Glycoside hydrolase 39, Lignocellulose, β-xylosidase

## Abstract

**Background:**

There is a continued need for improved enzymes for industry. β-xylosidases are enzymes employed in a variety of industries and although many wild-type and engineered variants have been described, enzymes that are highly tolerant of the products produced by catalysis are not readily available and the fundamental mechanisms of tolerance are not well understood.

**Results:**

Screening of a metagenomic library constructed of mDNA isolated from horse manure compost for β-xylosidase activity identified 26 positive hits. The fosmid clones were sequenced and bioinformatic analysis performed to identity putative β-xylosidases. Based on the novelty of its amino acid sequence and potential thermostability one enzyme (XylP81) was selected for expression and further characterization. XylP81 belongs to the family 39 β-xylosidases, a comparatively rarely found and characterized GH family. The enzyme displayed biochemical characteristics (K_M_—5.3 mM; V_max_—122 U/mg; k_cat_—107; T_opt_—50 °C; pH_opt_—6) comparable to previously characterized glycoside hydrolase family 39 (GH39) β-xylosidases and despite nucleotide identity to thermophilic species, the enzyme displayed only moderate thermostability with a half-life of 32 min at 60 °C. Apart from acting on substrates predicted for β-xylosidase (xylobiose and 4-nitrophenyl-β-D-xylopyranoside) the enzyme also displayed measurable α-L-arabainofuranosidase, β-galactosidase and β-glucosidase activity. A remarkable feature of this enzyme is its ability to tolerate high concentrations of xylose with a K_i_ of 1.33 M, a feature that is highly desirable for commercial applications.

**Conclusions:**

Here we describe a novel β-xylosidase from a poorly studied glycosyl hydrolase family (GH39) which despite having overall kinetic properties similar to other bacterial GH39 β-xylosidases, displays unusually high product tolerance. This trait is shared with only one other member of the GH39 family, the recently described β-xylosidases from *Dictyoglomus thermophilum*. This feature should allow its use as starting material for engineering of an enzyme that may prove useful to industry and should assist in the fundamental understanding of the mechanism by which glycosyl hydrolases evolve product tolerance.

**Supplementary Information:**

The online version contains supplementary material available at 10.1186/s12896-021-00722-6.

## Background

Lignocellulosic plant biomass could be a cheap and abundant feedstock for applications including biofuel production, bioplastics, the paper and pulp industry as well as pharmaceutical production [1–3]. The three main components of plant biomass are cellulose, hemicellulose, and lignin of which hemicellulose and lignin can take a variety of chemical forms depending on the plant species and, in general, are the plant cell wall components most recalcitrant to hydrolysis. Enzymatic degradation, following physicochemical disruption (steam explosion, high/low pH treatment or solubilization with ionic liquids), could offer a more efficient, environmentally friendly approach to assist in the degradation of plant biomass. Much research has focused on the discovery of more efficient enzymes to degrade cellulose and hemicellulose to fermentable sugars. Additionally, there is a need to discover enzymes with improved tolerance to industrial process conditions such as tolerance to hydrolysis by-products (inhibitors such as furfural, hydroxymethyl furfural), end products (glucose and xylose) and solvents [4]. Thermostable enzymes have the added advantage that they can retain activity at high temperatures reducing the need to cool feedstocks following physicochemical treatment to open the plant structure, prior to enzymatic hydrolysis [5]. The complete breakdown of lignocellulose requires the consortium of microorganisms which produce cellulases, hemicellulases and ligninases. In industrial processes, this is achieved through enzymatic cocktails which contain different enzymes that act on different components of lignocellulosic substrates [6]. Multi-substrate enzymes which hydrolyse different lignocellulosic substrates could offer a cost-effective fermentation process. The discovery of novel enzymes that might meet the needs of industry can be limited by traditional culture-based or mutation techniques [7]. Metagenomics is the direct interrogation of total DNA from an environmental sample without the need for culturing of the host organism. This tool provides a powerful approach for functional screening and identification of novel biocatalysts from unculturable or uncultured microorganisms from any environment [8].

Hemicellulose is a heteropolymer consisting of β-1,4 linked xylose monomers (xylan) as a major component with either arabinose, glucuronic acid, acetyl, feruloyl, and p-coumaryl side chain groups depending on the source material [9]. Hydrolysis of the xylan backbone requires the action of multiple enzymes which include xylanase (EC 3.2.1.8) and β-xylosidase (EC 3.2.1.27) that attack the xylan backbone, while side chain hydrolysis requires α-L-arabinofuranosidases (EC 3.2.1.55) and glucuronidase (EC 3.2.1.34) as they make the xylan backbone accessible to degradation [10]. In industry, β-xylosidases catalyse the final rate limiting step to release fermentable sugars from hemicellulose for use by microorganisms in large scale fermentations [11]. These applications include deinking of recycled paper [12], processing wood pulp to improve bleachability and brightness [13, 14], improving bread dough baking and nutritional quality [15], reducing the bitter flavour caused by xylosylated compounds in grape juice during extraction and liberation of aroma derived from xylosylated compounds of grapes during wine making [16] as well as hydrolysis of xylan to D-xylose for reduction to xylitol [17]. β-xylosidases belong to several glycoside hydrolase families (GH3, GH30, GH39, GH43, GH53, GH54, GH116 and GH120) with all of them functioning as retaining hydrolases except the GH43 family which are inverting enzymes [18]. Only 12 GH39 β-xylosidases enzymes present in the CAZY database have been characterized and of these few have been identified through metagenomic screens [19–23], making these rather rare enzymes. Patenting of at least one of these enzymes suggest some usefulness in certain applications (WO/2018/185150). Here, we report the basic biochemical characterization of a novel GH39 family β-xylosidase (XylP81) identified through functional screening of a horse manure compost derived metagenomic library.

## Results and discussion

### Metagenomic library screening, sequence identification and analysis

**Fig. 1:**
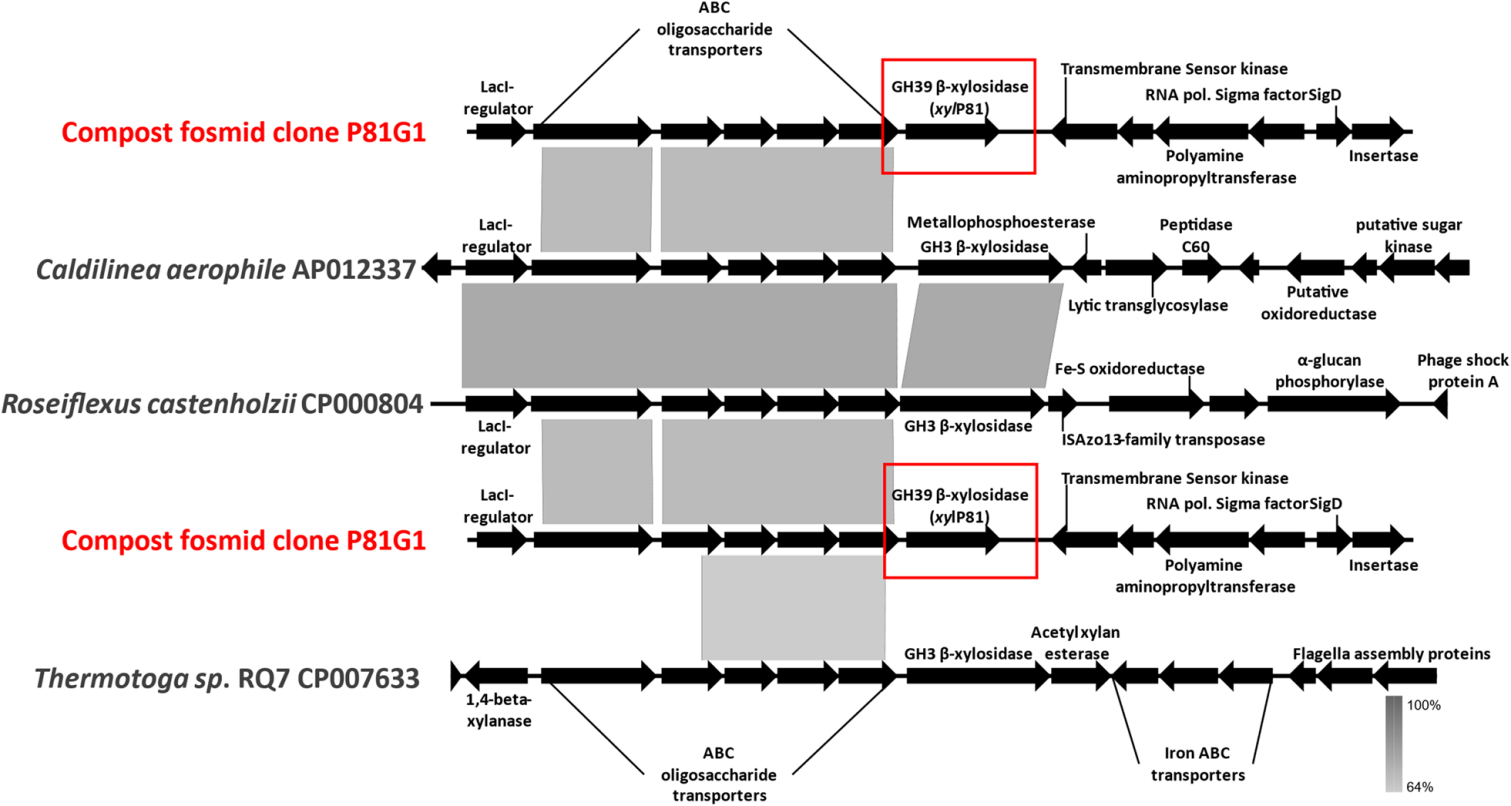
BLASTn comparison of the P81G1 fosmid insert to three genomic fragments from *Caldilinea aerophila* DSM 14535, *Roseiflexus castenholzii* DSM 13941 and *Thermotoga sp.* RQ7 respectively showing genomic synteny

A mDNA library of ~ 20,000 fosmid clones was generated and subjected to high throughput screening to detect β-xylosidase activity. A total of 26 positive hits were identified, and of these, the insert sequences for 18 clones that showed highest β-xylosidase activity were determined. The fosmid insert of clone P81G1 assembled as two fragments (15.34 kb and 12.10 kb). At the nucleotide level, a portion (8429 bp-14123 bp) of the 15 kb P81G1 insert was most similar to regions on the *Caldilinea aerophila* DSM 14535, *Roseiflexus castenholzii* DSM 13941 and *Thermotoga sp.* RQ7 genomes with 67% identity (Fig. 1). Although this region showed high similarity to oligopeptide and nickel transport systems, the region likely covers a putative oligosacchraride transport system as demonstrated for *Thermotoga maritima* [24–26]. The similarity at a nucleotide level to members of the *Chloroflexi* and other thermophilic species suggests that the DNA fragment may have originated from a thermophile. One ORF on this genomic fragment displayed clear similarity to a β-xylosidase (*xyl*P81; 1368 bp, 52.4 kDa) and no other easily identifiable glycoside hydrolases could be detected. Apart from the xylosidase located immediately downstream of these transporters, there appears to be no further synteny between these genomic layouts. The amino acid sequence coded for by *xyl*P81 was most similar to an “AraC family transcriptional regulator *Chloroflexi* bacterium” (HDU42522; Id. 80%: 382aa/Pos. 88%: 424aa), again showing the limitations of automated annotation, while the closest hit to an annotated β-xylosidase was a β-xylosidase from an *Anaerolineaceae* bacterium (MAU09869; Id. 75%: 381aa/Pos. 85%: 433aa). The absence of a secretion signal together with being found in an operon with putative xylooligosaccharide transporters under control of a LacI-like regulator, argues for this xylosidase being intracellular. The dbCAN2 server classified XylP81 as belonging to the GH39 family. Modelling and alignment of the XylP81 sequence with characterized GH39 enzymes showed that the catalytic residues (Glu162, His235 and Glu284; XylP81 numbering) are conserved as for the xylosidases from this family (Additional file 1: Fig. S3B). The residues proposed to be involved in substrate recognition (His60, Phe117, Asn161, Phe168, Tyr237, Trp322, Phe328, Glu330; XylP81 numbering) were also conserved, except for Tyr283 replaced by a proline (Pro289) in XylP81. Modelling shows that His241, part of the β-hairpin catalytic loop and not present in other GH39 β-xylosidases, overlaps the position taken by Tyr283 possibly substituting for this residue. As noted elsewhere [27, 28], overall, the catalytic site and substrate binding pockets are highly conserved, and these structures align very well. Thus, it should be expected that catalysis by XylP81 also operates by the catalytic mechanism (double displacement) described for GsXynB1 from *G. stearothermophilus* and TsXynB from *Thermoanaerobacterium saccharolyticum* [29–31].

The conservation of most of the catalytic and substrate recognition residues made the enzyme less appealing to characterize as the biochemical characteristics might be expected to be the same as for previously characterized enzymes. However, overall amino acid identity and similarity compared with characterized GH39 xylosidases showed highest identity (34%) and similarity (58%) to enzyme to TsXynB. Additionally, phylogenetic assessment (Additional file 1: Fig. S2), demonstrated that this enzyme occupied a unique position in the tree and did not cluster closely with previously characterized GH39 representatives. Through this analysis it also became apparent that the “xyl3” enzyme described by [21] belongs to the GH39 family as opposed to the GH1 family. The unique sequence space occupied by the enzyme, together with its prospective thermophilic origins, prompted us to investigate XylP81 further.

### Biochemical characterization of XylP81

#### Thermostability, temperature and pH optimum

The recombinant XylP81 protein displayed a broad temperature optimum profile with highest activity at 50 °C (Fig. 3a). Although several thermostable β-xylosidases have been described from this family, GH39 enzymes do not exclusively derive from thermophiles. XylP81 is a moderately thermostable enzyme as it retains 90% activity after 1-h incubation at 50 °C with a half-life of 32 min at 60 °C (Fig. 3e). According to the equilibrium model for the effect of temperature on enzyme activity, a broad temperature optimum is likely due to a smaller change in enthalpy (Δ*H*_*eq*_) during the transition from active enzyme to inactive but non-denatured enzyme [32]. The two parameters Δ*H*_*eq*_ and *Teq*, which is defined as the temperature at the midpoint of the transition between active and non-denatured inactive enzyme, are properties of the active site and the effect of various substrates on these parameters for particular enzymes have been demonstrated [33]. Different substrates can lead to changes in Δ*H*_*eq*_ which results in broadening of the *T*_*opt*_ profile likely due to stabilization of the active site in the presence of various substrates. The temperature optimum profile may therefore partly be the result of the substrate used, and we note that several GH39 β-xylosidases display broad temperature optima on pNPX. Inspection of the primary sequence showed that the C-terminal extension present in most GH39 β-xylosidases from extremophiles was not present [27, 28, 31]. This suggests that despite the overall sequence similarity to members of the *Chloroflexi* and other thermophilic genera, the enzyme likely derives from a mesophile. It also suggests that the enzyme is monomeric in solution as the C-terminal extension is thought to be the primary reason for tetramer formation in those enzymes which have this quaternary structure [28, 34]. The pH optimum is 6, similar to the optima reported for other GH39 β-xylosidases (pH5—pH7.5) with a bell-shaped curve indicating two titratable groups at pKa values of ~ 4.6 and 7.4 [30].

### Substrate specificity and kinetics

**Fig. 2:**
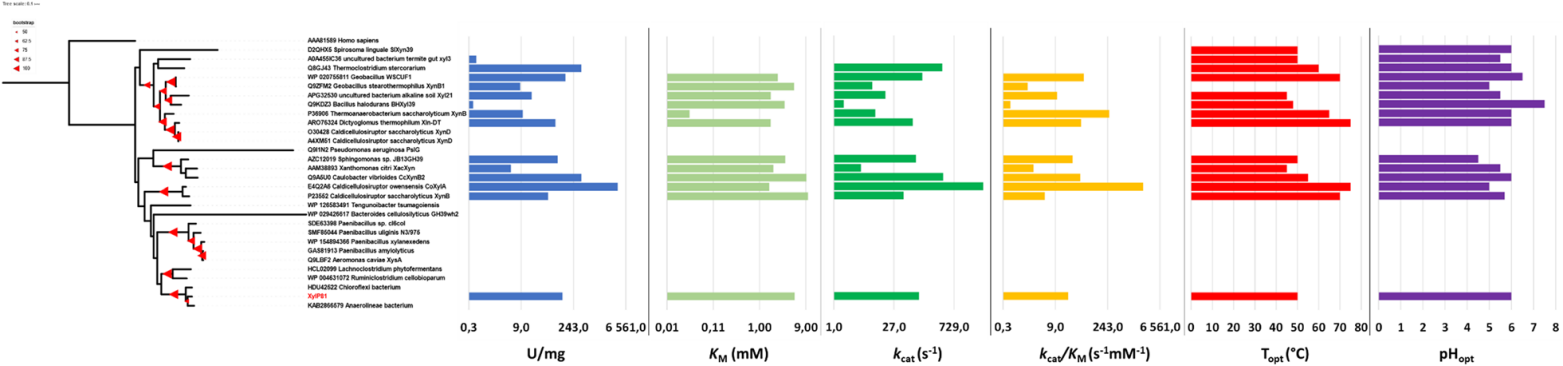
Phylogenetic assessment and comparison of biochemical parameters for XylP81 with characterized members of the GH39 family. T_opt_ and pH_opt_ data are displayed as raw values. Red triangles indicate bootstrap replicates between 50 and 100%

**Fig. 3:**
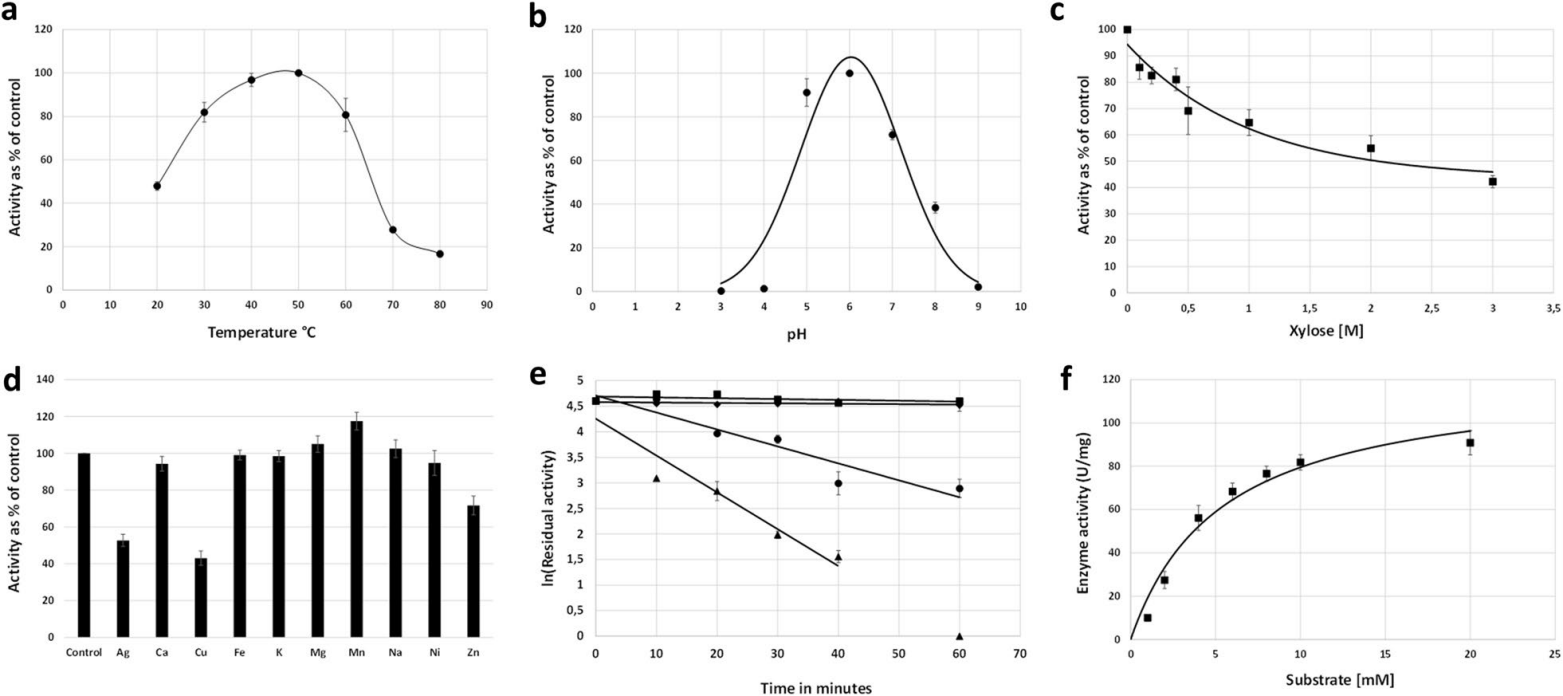
Summary of biochemical characteristics of XylP81: **A** Temperature optimum; **B** pH optimum; **C** Xylose inhibition; **D** Sensitivity to metal ions; **E** Thermostability (*black triangle*—70 °C, *black circle*—60 °C, *black square*—55 °C, *black diamond*—50 °C); **F** Michaelis curve using pNPX as substrate

The GH39 family is known to display two main activities, β-xylosidase and α-iduronidase, and a multitude of secondary activities (transglycosylase, α-arabinofuranosidase, β-glucosidase and PslG’s unique activity). XylP81 showed high specific activity on pNPX (122 U/mg) with minor secondary activities including α-arabinofuranosidase, β-galactosidase and low but detectable β-glucosidase activity (Additional file 1: Table S1). It also had detectable activity on beechwood- and birchwood xylan indicating low endoxylanase activity, similar to that described for *Geobacillus* WSCUF-1 [35]. The small quantity of reducing sugar detected likely comes from the degradation of small amounts of xylooligosaccharides present in the substrate. Modelling of the XylP81 sequence on the structure of XacXynB (QMEAN − 3.80), and comparison of the XylP81 model with the structures of CcXynB2, TsXynB and GsXynB1 (Additional file 1: Fig. S3) showed that, like CcXynB2 and XacXynB, XylP81 has the longer α-helix-containing loop from the auxiliary domain (Ser399-Glu419) that interacts with the catalytic β-hairpin forcing it to adopt an open conformation [28, 34]. The *K*_M_ for XylP81 on pNPX was 5.3 mM and is in line with what has been reported for most bacterial GH39 xylosidases (Figs. 2 and 3f). Two other notable differences between XylP81 and related enzymes are Thr166 and Val167 which are either Lys/Val/Asp or Asn/Glu/Gly/Asp in the other structures, respectively. These residues located at the mouth of the substrate binding pocket may modify the affinity for natural substrates and may be the reason for the range of *K*_M_ values observed among them. In XacXynB substitution of both positions with the corresponding residues found in CcXynB2 (K166D and D167G) resulted in higher *K*_M_ values, which was unexpected [28]. The catalytic β-hairpin is a highly flexible structure [28] capable of interacting with substrate molecules and is a part of the protein that has high variability with few conserved residues. These two elements may work in unison to form a thumb and forefinger arrangement which sense and clamp the substrate thereby contributing to the *K*_M_ for a particular substrate. The *k*_cat_ for GH39 β-xylosidases spans a wide range covering several orders of magnitude owing predominantly to vastly different specific activities whereas, except for TsXynB, the *K*_M_ values are within an order of magnitude (Fig. 2). It therefore seems counter intuitive that the residues responsible for *k*_cat_ in the catalytic site are so highly conserved yet the specific activities and *k*_cat_ values span such a wide range, whereas the residues responsible for *K*_M_ are a mixture of highly variable and conserved residues, yet their values are far closer. This may argue for a dominant role of the conserved resides in determining *K*_M_. The exceptionally low *K*_M_ observed for TsXynB could be a consequence of the affinity of its catalytic β-hairpin for pNPX promoting formation of the Michaelis complex. The radical variability of *k*_cat_ against the backdrop of highly conserved catalytic residues needs further investigation through mutation and QM/MM simulations [36].

### Product tolerance

XylP81 shows remarkable tolerance to xylose as it retains ~ 40% activity in the presence of 3 M xylose (*K*_i_—1.33 M), a feature shared with the recently characterized GH39 β-xylosidase from *Dictytoglomus thermophilum*, Xln-DT [37] (Fig. 3c). This is not a feature of the GH39 family that has been extensively explored and the molecular mechanisms by which other glycosyl hydrolases are either resistant, tolerant or stimulated by their end products is also not yet understood. A study by [38] looking at glucose tolerance in a metagenome-derived GH1 family β-glucosidase suggested that subsites in the channel leading to the catalytic pocket have an affinity for glucose. The titration of glucose molecules to subsites instead of the catalytic site is proposed to allow the enzyme to operate in the presence of even high concentrations of product. A number of phenomena such as an increase in *K*_M_ with an increase in product concentration or stabilization of the enzyme may all be invoked to explain the high product tolerance [39, 40]. Although not determined here, high transxylosylation activity could be the reason for apparent high product tolerance, where XylP81 preferentially uses xylose instead of water as an acceptor for the xylosyl moiety during the catalytic degradation of pNPX [41, 42]. Transxylosylation is likely also favoured under high product concentrations. The enzyme from *Geobacillus* WSCUF-1 has been assayed for its tolerance to product inhibition and unlike XylP81, retained only 50% activity at 300 mM xylose [35]. Although the crystal structure for the GH39 β-xylosidase from *Geobacillus* WSCUF-1 is not available the structure for the very closely related GsXynB1 has been solved [27]. Comparison of the structure from GsXynB1 with models of XylP81 and the Xln-DT enzyme did not offer any immediate clues as to the nature of improved product tolerance by the latter enzymes. A notable difference between XylP81 and published structures of GH39 β-xylosidases is an additional 5 amino acid insertion from residue 192 to 198 also present in the *Chloroflexi* sp. and *Anaerolineae* sp. enzymes which appears to close off one side of the active site cleft (Additional file 1: Fig. S4).

### Sensitivity to metal species

When testing the effect of cations on the activity of XylP81, it was most affected by Cu^2+^ and Ag^2+^ with a 57% and 48% decrease in activity respectively following incubation in the presence of these metal salts with slight improvement (15%) on incubation with Mn^2+^ (Fig. 3d). Similarly, three other GH39 β-xylosidaes (*Geobacillus* WSUCF-1, Xln-DT and CcXynB2) were reported to lose between 60 and 77% activity in the presence of Cu^2+^ [35, 42–44]. Xln-DT displayed 15% increase in activity on incubation with 5 mM Mn^2+^ whereas there was no improvement at 1 mM. CcXynB2 activity was reduced (33%) when incubated with 2 mM Mn^2+^ while the *Geobacillus* WSUCF-1 enzyme showed no improved activity when assayed at 1 mM Mn^2+^. For JB13GH39 Cu^2+^ had a positive effect on activity as did Mn^2+^ with a 15% improvement while for Xyl21 both these metal ions reduced activity [23, 42]. The large negative electrostatic potential observed for the active sites of these enzymes should attract positively charged ions [31]. The increase in activity with Mn^2+^ is in line with the expected role for this non-redox-active metal which likely makes functional groups more electrophilic (more acidic) or stabilizes charged intermediates/transition states [45]. Coordination of a cupric ion (Cu^2+^), the most competitive metal in the Irving-Williams series, by glutamate and histidine residues in the active site may be responsible for the reduced enzyme activity in the presence of this metal [46]. Additionally, differences in co-ordination geometry may be responsible for the differences in activity between different metal species, as has been observed in other enzymes such as GlxI from *Clostridium acetobutylicum* [47]. These metals should not pose a problem for the enzyme i*n vivo* as the cytoplasm is a metal-regulated environment but may have implications for their use in commercial settings.

## Conclusion

Here we describe a novel GH39 β-xylosidase isolated from a compost metagenome. Comparison of its basic biochemical characteristics to those that have been described shows just how remarkably flexible amino acid compositions can be yet resulting in highly similar enzymatic activities. The high tolerance of XylP81 and Xln-DT to xylose should allow for the identification of the features that enable this and make these enzymes good starting points for engineering of enzymes that may be commercially desirable.

## Materials and methods

### Metagenome library construction, screening, sequence identification and phylogenetic assessment

Horse manure compost was collected in March 2013 from a commercial compost farm (Master Organics, Philippi, Cape Town) located in the Western Cape Province of South Africa (− 34.048340, 18.529347). The compost source material consisted of an unspecified mix primarily composed of horse manure, wood chips and sawdust with a maximum measured temperature of 70 °C. Metagenomic DNA (mDNA) was extracted using the chemical lysis method as previously described [48]. Each extraction was performed using 1.6 g compost material re-suspended in 5 ml extraction buffer (100 mM Tris–HCl, pH 8.0; 100 mM EDTA, pH 8.0; 100 mM sodium phosphate, pH 8.0; 1.5 M NaCl; 1% CTAB). A volume of 20 μl proteinase K (10U) was added and the mixture incubated at 37 °C for 30 min. Cell lysis was performed through the addition of SDS (2% w/v) and PVPP added to a final concentration of 0.5% w/v. The sample was further incubated at 65 °C for 2 h. Debris was pelleted by centrifugation at 6000×*g* for 10 min at room temperature. The supernatant was carefully removed and added to 1 volume phenol:chloroform:isoamyl alcohol (25:24:1) followed by gentle inversion of the tube and centrifugation at 16,000×*g* for 10 min. The aqueous phase was removed, added to an equal volume of chloroform and transferred to micro-centrifuge tubes. Following centrifugation at 16,000×*g* for 10 min, the aqueous phase was again recovered, and nucleic acid was precipitated with 1 volume 100% isopropanol at room temperature overnight. Nucleic acids were pelleted by centrifugation at 16,000×*g* for 20 min at room temperature. The supernatant was discarded, and the pellet was washed with ice cold 70% v/v ethanol, air-dried and re-suspended in an appropriate volume of 1 × TE buffer. The extracted DNA was further purified as previously described [49]. Briefly, 500 μl of mDNA was mixed with 500 μl of 2% molten (55 °C) agarose gel prepared in TAE buffer. The mixture was allowed to solidify in a 1 ml plastic syringe of which the tip was cut off. The solidified agarose-plug containing compressed metagenomic DNA was removed from the syringe and placed in a 15 ml centrifuge tube containing 80% formamide and 0.8 M NaCl in 20 mM Tris–HCl buffer (pH 8.0). The plug was washed by gently inverting the tube several times for 1 h. Following incubation, the formamide solution was replaced with fresh solution, and the plug was incubated overnight at room temperature with gentle agitation. The agarose plug was embedded at the top of a 1% low meting point (LMP) agarose gel and mDNA was electrophoresed for 3 h at 70 V. High-molecular weight (> 23 kb) DNA was excised from the LMP agarose. The agarose plug was transferred to a sterile 2 ml microfuge tube and incubated at 70 °C for 10 min to melt the agarose. This was followed by additional incubation at 42 °C to equilibrate the mixture. DNA-agarose mixture was treated with agarase (Fermentas) at 1U of enzyme per 10 mg of agarose followed by gentle mixing and incubation at 42 °C for 2 h. The reaction was heat inactivated at 70 °C for 10 min followed by centrifugation at 9000×*g* for 10 min. Supernatant was removed, and DNA was precipitated by adding 2.5 volumes of absolute ethanol and 0.1 volume of sodium acetate (pH 7.0), followed by incubation at − 20 °C overnight. mDNA was pelleted by centrifugation at 16,000×*g* for 30 min.

An mDNA library was constructed using the CopyControl™ FosmidLibrary production kit (Epicentre) according to the manufacturer’s guidelines. Extracted high-molecular weight mDNA was end-repaired using End-It™ DNA end repair kit (Epicentre) and purified by Phenol:Chloroform:Isoamyl extraction followed by EtOH/Na-Acetate precipitation overnight at − 20 °C. Following purification, the DNA was ligated to the CopyControl pCC1Fos™ vector and packaged using MaxPlax™ Lambda phage extract. Following transfection into *E. coli*-EPI300-T1R the library titre was determined and the library diluted to produce aliquots of 1 ml with ~ 1000 transformants per aliquot. These were stored at − 80 °C until use.

Approximately one thousand clones were plated per Q-tray (Corning Inc) and a Genetix QPix2-XT automated colony picker was used to transfer individual clones into 96-well microplates, with each well containing 50 μl LB broth supplemented with 12.5 μg/ml chloramphenicol, 2.5 mg/ml pNPX and 0.02% (w/v) L-arabinose for fosmid copy number amplification. The microplates were sealed with breathable sealing membrane (Sigma, USA) and cultured at 37 °C overnight with shaking. Positive clones were identified by change of LB broth colour to orange/yellow. Following identification of positive clones, glycerol was added to a final concentration of 20% v/v and the plates were stored at − 80 °C.

Insert DNA sequences for fosmids of interest was determined through next generation sequencing using an Illumina MiSeq. Sequencing libraries were prepared using the Nextera XT library prep kit and sequenced using a V2 500cycle reagent kit with a 15% phiX spike as per manufacturers’ recommendation. This resulted in paired end sequences (2 × 250 bp). Sequences were assembled using CLC Genomics Workbench version 7.5.1. and contigs were annotated using Prokka v1.1.2 [50] through the KBase [51] online analysis platform.

Genomic synteny and nucleotide level comparison of contig P81G1 with related sequences was performed using Easyfig with the following parameters: Min identity value 20, E-value 1 × 10^−10^ and Min length 40 [52]. The deduced protein sequence of XylP81 was analysed using the dbCAN2 server [53] to identify the glycoside hydrolase (GH) family. Multiple sequence alignments were performed using MUSCLE, viewed and edited in AliView [54, 55]. Phylogenetic inference was performed according to [56] using the amino acid sequences of characterized bacterial and archaeal enzymes in the CAZY database together with selected top BLASTp hits from the NCBI database (uncharacterized enzymes in Fig. 3). For XylP81 related sequences the AraC-like domain, found at the N-terminal of some GH39 xylosidases was removed, prior to alignment. Maximum likelihood trees were constructed using PHYML on the ATGC server (http://www.atgc-montpellier.fr/phyml/). Trees were visualized and annotated using iTOL [57]. Modelling of the XylP81 structure was performed using SWISS-MODEL (https://swissmodel.expasy.org/) with the *Xanthomonas axonopodis* pv. citri GH39 β-xylosidase XacXynB structure (6uqj) as template (Additional file 1: Figure S3) and structures visualized in PyMol 2.0.7 (Schrödinger, LLC).

### Expression vector construction, protein expression and purification

The P81 gene product was synthesized and cloned into pET21a(+) by Biomatik (https://www.biomatik.com/). For protein production, inoculated broth cultures (50 ml in a 250 ml flask) were cultured at 37 °C until OD_600nm_ of 0.5–0.6 was reached. Protein expression was induced through addition of isopropyl-β-D-thiogalactopyranoside (IPTG) to a final concentration of 0.5 mM and cultured overnight at 37 °C with shaking (150 rpm). Cells were harvested by centrifugation at 4 °C, 3265×*g* for 5 min. The pellet was resuspended in 20 mM Tris–HCl (pH 7.9), 50 mM NaCl and sonicated on ice using a Bandelin Sonopuls HD 2070 (58% power, 5 cycles for 30 s). The sonicated cell debris was collected by centrifugation at 4 °C, 13 000×*g* for 20 min. The soluble fraction was filtered through a 0.45 µm syringe filter and purified using nickel affinity chromatography. The purity of the protein was analysed on a 12% SDS-PAGE stained with Coomassie (Additional file 1: Figure S1). The protein was concentrated using an Amicon® Ultra-15 centrifugal filter device with a 50 kDa nominal molecular weight cut off and the concentration determined using the Bradford method [58].

### Enzyme assays

The hydrolysis of chromogenic substrates pNPX (Sigma Aldrich) was used to measure the enzyme activity of XylP81. The standard reaction mixture consisted of 240 µL of 2 mM substrate in 50 mM sodium phosphate buffer (pH 6) and 0.5 µg of purified enzyme. The reaction was incubated at 37 °C for 20 min and terminated by the addition of 1 mL, 1 M Na_2_CO_3._ The amount of p-nitrophenol product was determined by measuring the absorbance at 410 nm using a microplate spectrophotometer (SPECTROstar Nano, BMG LabTech UK). One unit (U) of enzyme activity was defined as the amount of enzyme that releases 1 µmol of p-nitrophenol per minute per milligram of protein.

The optimum pH for both enzymes was determined in a pH range of 3–9 using three different buffer systems under standard assay conditions. The buffers used were 50 mM citrate phosphate buffer (pH 3–5), 50 mM sodium phosphate (pH 6 and 7) and 50 mM Tris–HCl (pH 8 and 9). A Gaussian distribution was fit to this data to determine the pH_opt_. The optimum temperature (T_opt_) was determined by incubation of the enzyme reaction at various temperatures (30, 40, 50, 60, 70 and 80 °C) in 50 mM sodium phosphate (pH 6) and enzyme activity was measured as described above. The thermostability of the enzyme was determined by incubation of the enzymes at 50 °C, 55 °C, 60 °C and 70 °C for 1 h. An aliquot of enzyme was removed and assayed for activity every 10 min until the 1-h time point was reached at each temperature. The effect of xylose on the activity of XylP81 was evaluated by assaying the enzyme in the presence of various concentrations of the xylose (0.1 M, 0.2 M, 0.4 M, 0.5 M, 1 M, 2 M, and 3 M). A one phase decay curve was fit to this data in GraphPad Prism version 8.2.1 (GraphPad Software, Inc., San Diego, CA, USA). The effect of metal ions was evaluated by measuring enzyme activity of XylP81 with the addition of metal salts into the reaction mixture at a final concentration of 5 mM. The sulphate salts of all metals were used with the exception of AgNO_3_, MnCl_2_, KCl and Ca(NO_3_)_2_.

To generate the Michaelis curve initial velocity of enzyme reactions was measured by assaying substrate concentrations of 1 mM–20 mM for 20 min with a constant enzyme concentration of 0.5 µg under standard assay conditions using pNPX as substrate. Enzyme kinetic parameters were determined by non-linear regression curve fitting of the Michaelis–Menten equation.

The substrate specificity was investigated using different chromogenic pNP-linked substrates which included pNPG, pNPX, pNPA, and pNPGal. The activity of the purified enzymes was also determined on various polysaccharides (beechwood xylan, birchwood xylan) by measuring reducing sugars using the DNS method [59]. Briefly, 10 µg of enzyme was incubated with 900 µL 50 mM sodium phosphate buffer at (pH 6) with substrate (1% w/v). The reaction was incubated for 30 min at 50 °C and terminated by the addition of 1.5 mL DNS reagent and boiled for 10 min. The absorbance was measured at 540 nm and the amount of reducing sugars and specific activity was determined from standard curves of xylose and glucose.

## Supplementary Information

**Additional file 1:** **Figure S1.** SDS-PAGE analysis XylP81 expression. A) Lane M: ColorPlus prestained protein ladder, broad range (10-230 kDa); lane 1: E. coli-pET21a no insert uninduced; lane 2: E. coli-pETP81 uninduced soluble fraction; lane 3: E. coli-pETP81 induced soluble fraction; lane 4: E. coli-pET21a no insert induced; lane 5: E. coli-pETP81 uninduced insoluble fraction; lane 6: E. coli-pETP81 induced insoluble fraction. B) Purified XylP81 following metal affinity chromatography purification. lane M: ColorPlus prestained protein ladder. **Figure S2.** Unrooted maximum likelihood phylogenetic tree of characterized GH39 amino acid sequences including XylP81, excluding unchracterized and sequences closely related to XylP81. **Figure S3.** A) Alignment of the structures for TsXynB (magenta) and GsXynB1 (blue) with a model of XylP81(light green) B) Alignment of all published GH39 β-Xylosidase structures with a model of XylP81 based on the Xanthomonas axonopodis pv. citri structure (6uqj). Conserved residues are shown as sticks. Those involved in substrate recognition are coloured orange and catalytic residues are coloured blue. **Figure S4.** Side view of the active site cleft of XacXynB (top panel) compared with that of XylP81 (bottom panel)showing the closed of cleft as a consequence of the extra five amino acids at the end of alpha helix 7. Modified conserved residues are highlighted in pink. **Table S1.** XylP81 substrate utilization compared with characterized GH39 β-xylosidases.

## Data Availability

The *xylP81* gene sequence is available on the Genbank database under accession number MN542424.1, and the gene could be made available on request from the authors.

## Abbreviations

DNS: Dinitrosalicylic acid
pNPX: 4-Nitrophenyl-β-D-xylopyranoside
pNPG: 4-Nitrophenyl-β-D-glucopyranoside
pNPA: 4-Nitrophenyl-α-L-arabinofuranoside
pNPGAL: 4-Nitrophenyl-β-D-galactopyranoside
kDa: Kilo dalton
