# Supplementary material for: Characterization of a highly xylose tolerant β-xylosidase isolated from high temperature horse manure compost
Source: BMC Biotechnol. 2021 Oct 24;21:61. doi: 10.1186/s12896-021-00722-6 (PMC8543862; doi:10.1186/s12896-021-00722-6)
Supplement: Supplementary file 1 — Additional file 1: Figure S1. SDS-PAGE analysis XylP81 expression. A) Lane M: ColorPlus prestained protein ladder, broad range (10-230 kDa); lane 1: E. coli-pET21a no insert uninduced; lane 2: E. coli-pETP81 uninduced soluble fraction; lane 3: E. coli-pETP81 induced soluble fraction; lane 4: E. coli-pET21a no insert induced; lane 5: E. coli-pETP81 uninduced insoluble fraction; lane 6: E. coli-pETP81 induced insoluble fraction. B) Purified XylP81 following metal affinity chromatography purification. lane M: ColorPlus prestained protein ladder. Figure S2. Unrooted maximum likelihood phylogenetic tree of characterized GH39 amino acid sequences including XylP81, excluding unchracterized and sequences closely related to XylP81. Figure S3. A) Alignment of the structures for TsXynB (magenta) and GsXynB1 (blue) with a model of XylP81(light green) B) Alignment of all published GH39 β-Xylosidase structures with a model of XylP81 based on the Xanthomonas axonopodis pv. citri structure (6uqj). Conserved residues are shown as sticks. Those involved in substrate recognition are coloured orange and catalytic residues are coloured blue. Figure S4. Side view of the active site cleft of XacXynB (top panel) compared with that of XylP81 (bottom panel)showing the closed of cleft as a consequence of the extra five amino acids at the end of alpha helix 7. Modified conserved residues are highlighted in pink. Table S1. XylP81 substrate utilization compared with characterized GH39 β-xylosidases. [file 12896_2021_722_MOESM1_ESM.docx]

**Characterization of a highly xylose tolerant β-xylosidase isolated from high temperature horse manure compost**

**Kanyisa Ndata^1^, Walter Nevondo^1,2^, Bongi Cekuse^1^, Leonardo Joaquim van Zyl^1^, Marla Trindade^1^**

^1^Institute for Microbial Biotechnology and Metagenomics, Department of Biotechnology, University of the Western Cape, Bellville, South Africa

^2^ Division of Medical Virology, Institute of Infectious Disease and Molecular Medicine, Faculty of Health Sciences, University of Cape Town, Cape Town, South Africa

Corresponding author: Leonardo Joaquim van Zyl; [lvanzyl@uwc.ac.za](mailto:lvanzyl@uwc.ac.za); +27219592325


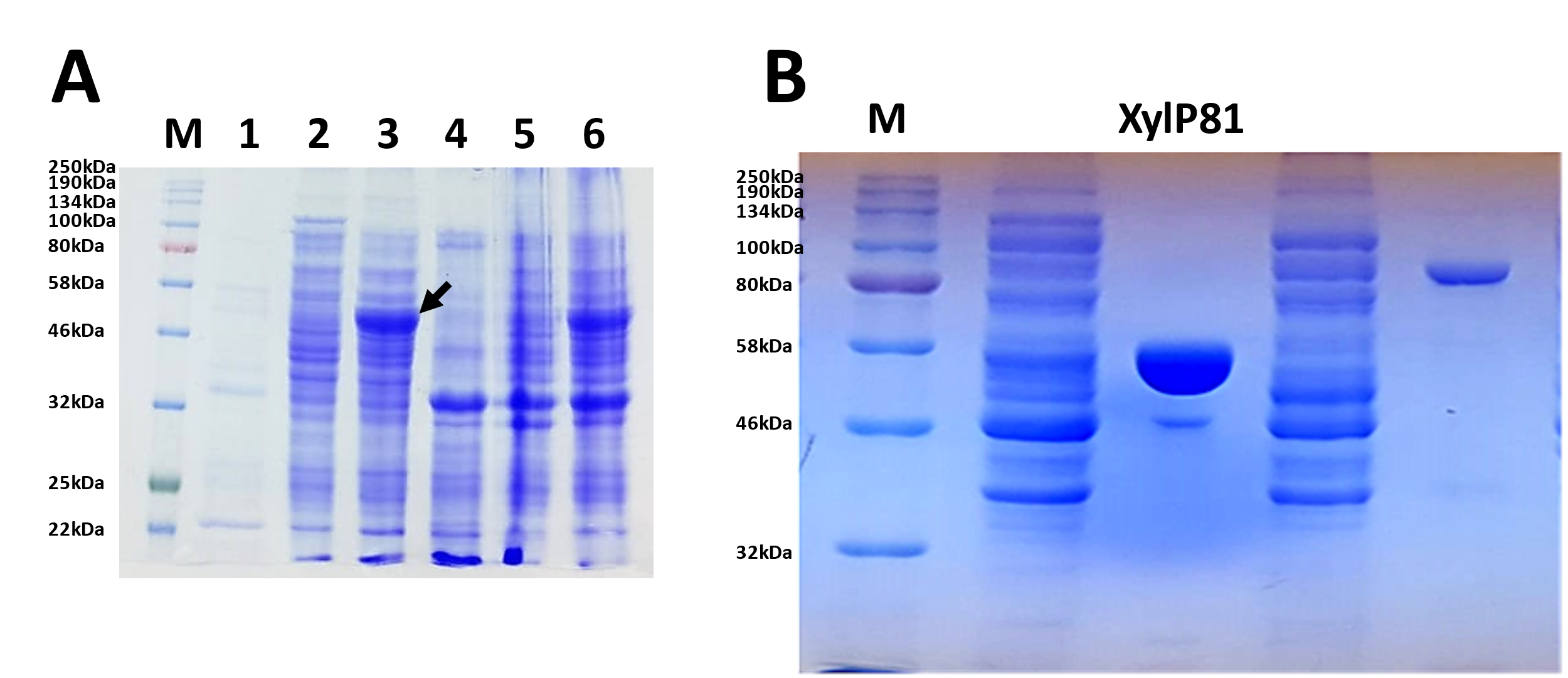


**Figure S1:** SDS-PAGE analysis XylP81 expression. A) Lane M: ColorPlus prestained protein ladder, broad range (10-230 kDa); lane 1: *E. coli*-pET21a no insert uninduced; lane 2: *E. coli*-pETP81 uninduced soluble fraction; lane 3: *E. coli*-pETP81 induced soluble fraction; lane 4: *E. coli*-pET21a no insert induced; lane 5: *E. coli*-pETP81 uninduced insoluble fraction; lane 6: *E. coli*-pETP81 induced insoluble fraction. B) Purified XylP81 and BglP89 following metal affinity chromatography purification. lane M: ColorPlus prestained protein ladder


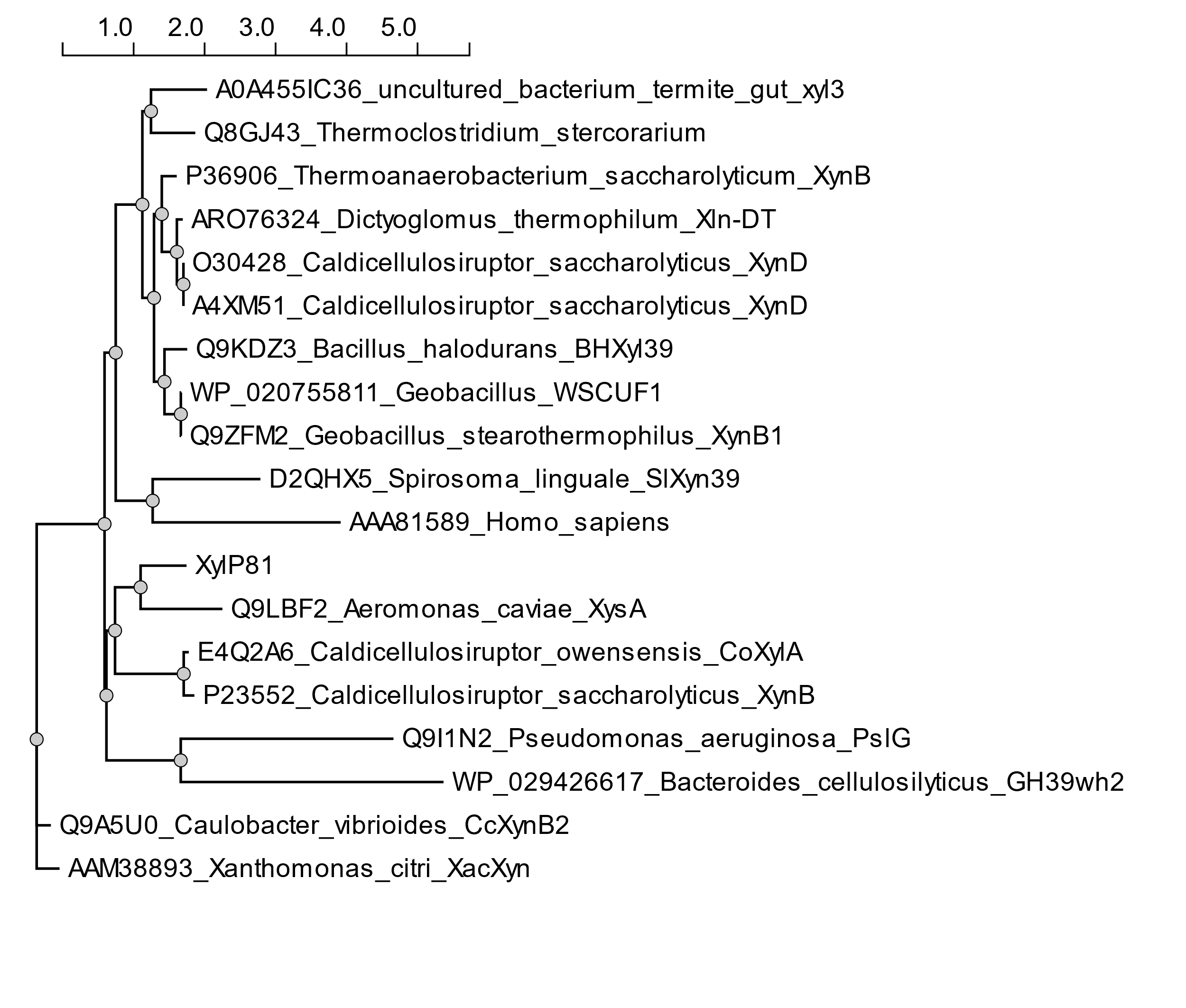


**Figure S2:** Unrooted maximum likelihood phylogenetic tree of characterized GH39 amino acid sequences including XylP81, excluding unchracterized and sequences closely related to XylP81


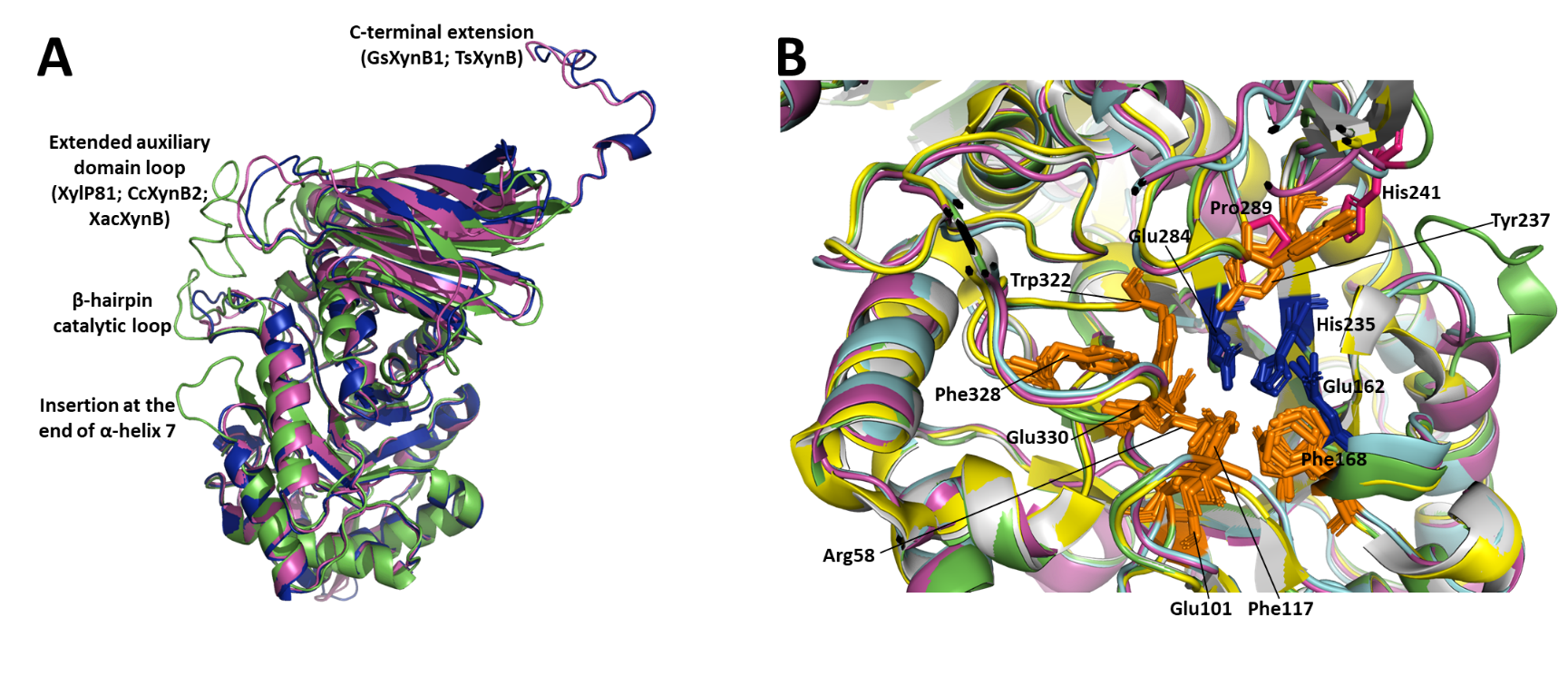
 **Figure S3: A)** Alignment of the structures for TsXynB (magenta) and GsXynB1 (blue) with a model of XylP81(light green) **B)** Alignment of all published GH39 β-Xylosidase structures with a model of XylP81 based on the Xanthomonas axonopodis pv. citri structure (6uqj). Conserved residues are shown as sticks. Those involved in substrate recognition are coloured orange and catalytic residues are coloured blue


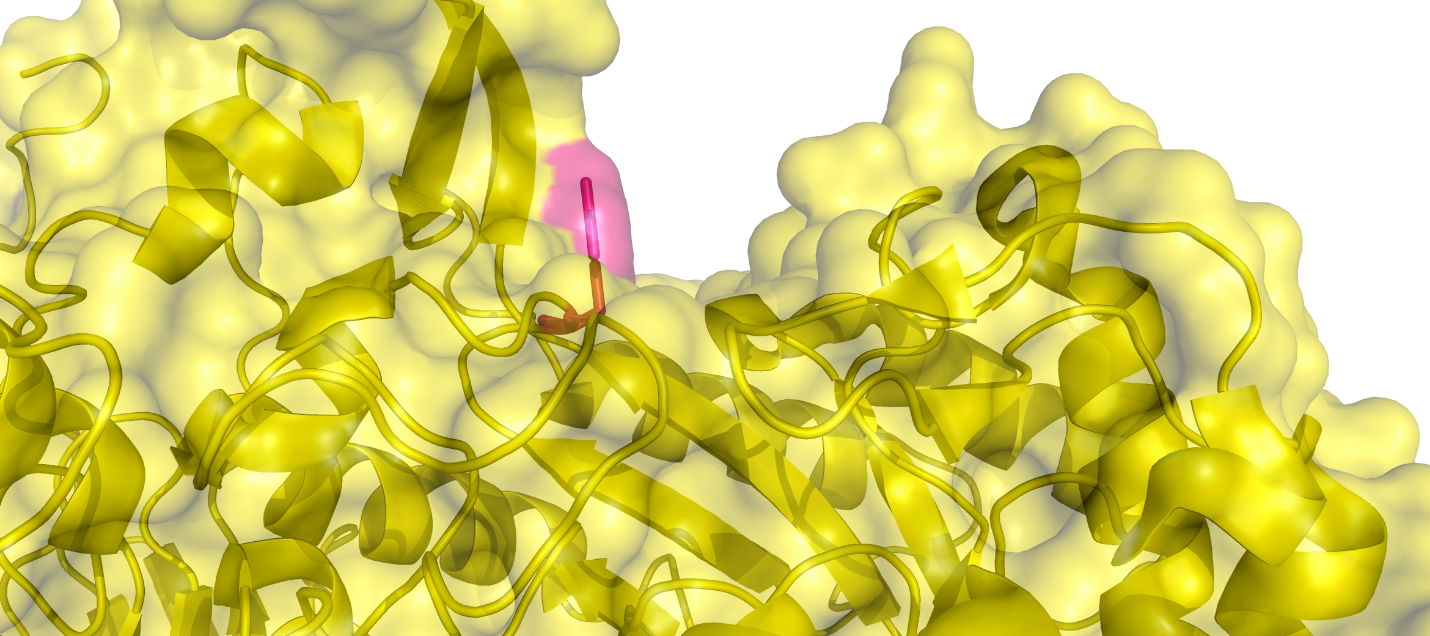


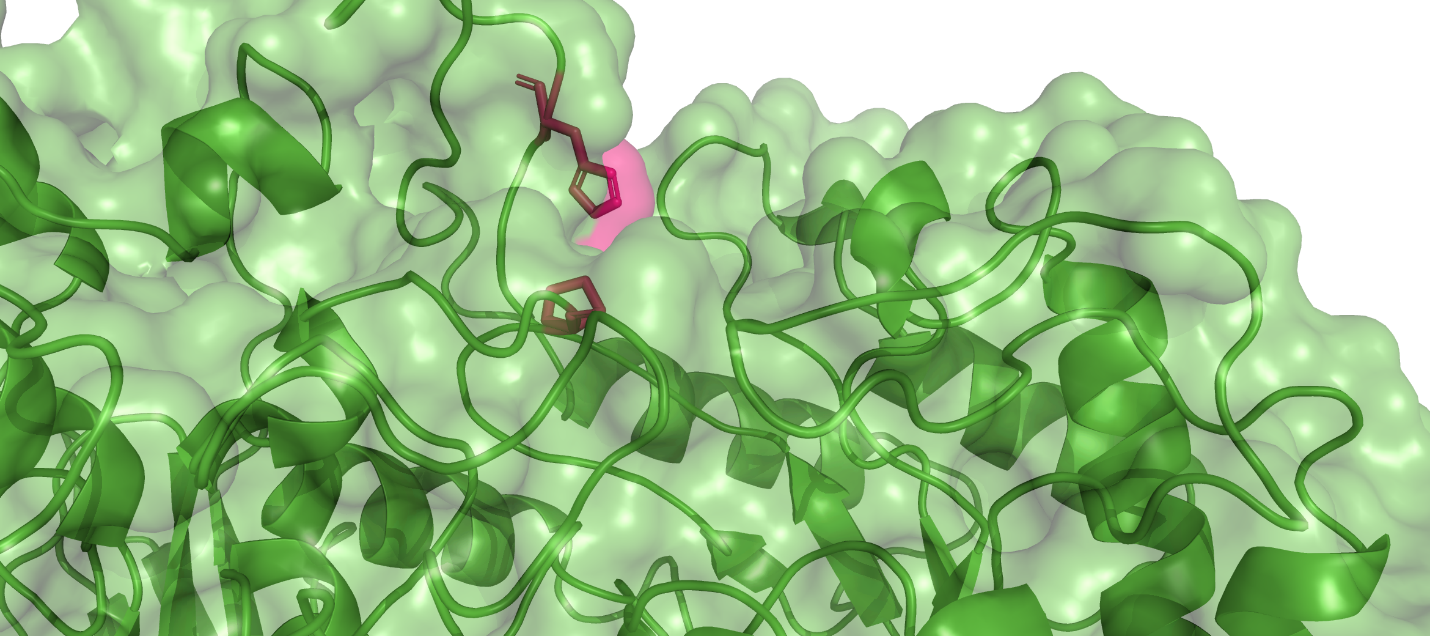


**Figure S4:** Side view of the active site cleft of XacXynB (top panel) compared with that of XylP81 (bottom panel) showing the closed of cleft as a consequence of the extra five amino acids at the end of alpha helix 7. Modified conserved residues are highlighted in pink

**Supplementary Table S1.** XylP81 substrate utilization compared with characterized GH39 β-xylosidases

| **Enzyme** | **Substrate** | **Specific activity (U/mg), % or Yes/No** | **Reference** |
| --- | --- | --- | --- |
| XylP81 | Xylobiose | Yes | This study |
|  | Xylotriose | Yes | This study |
|  | pNPX | 122 U/mg ± 4.5 | This study |
|  | pNPA | 17.4 U/mg ± 0.18 | This study |
|  | pNPGal | 3.6 U/mg ± 0.95 | This study |
|  | pNPG | 1.1 U/mg ± 1.16 | This study |
|  | Beechwood xylan | 0.0089 U/mg ± 0.003 | This study |
|  | Birchwood xylan | 0.04 U/mg ± 0.004 | This study |
| WSUCF1 | Xylobiose | Yes | 35 |
|  | Xylotriose | Yes | 35 |
|  | pNPX | 147 U/mg | 35 |
|  | pNPA | No | 35 |
|  | pNPGal | - |  |
|  | pNPG | No | 35 |
|  | Beechwood xylan | No | 35 |
|  | Birchwood xylan | No | 35 |
| Xln-DT | Xylobiose | Yes | 37 |
|  | Xylotriose | - |  |
|  | pNPX | 100% ± 2.01 | 37 |
|  | pNPA | No | 37 |
|  | pNPGal | No | 37 |
|  | pNPG | 23% ± 1.08 | 37 |
|  | Beechwood xylan | - |  |
|  | Birchwood xylan | - |  |
| Bxl39A | Xylobiose | Yes | 1 |
|  | Xylotriose | - |  |
|  | pNPX | 100% | 1 |
|  | pNPA | No | 1 |
|  | pNPGal | No | 1 |
|  | pNPG | No | 1 |
|  | Beechwood xylan | - |  |
|  | Birchwood xylan | - |  |
| CcXynB2 | Xylobiose | - |  |
|  | Xylotriose | - |  |
|  | pNPX | 76.7 U/mg | 44 |
|  | pNPA | - |  |
|  | pNPGal | - |  |
|  | pNPG | - |  |
|  | Beechwood xylan | - |  |
|  | Birchwood xylan | - |  |
| XylBH39 | Xylobiose | Yes | 60 |
|  | Xylotriose | No | 60 |
|  | pNPX | 8.61 U/mg | 60 |
|  | pNPA | No | 60 |
|  | pNPGal | No | 60 |
|  | pNPG | Low | 60 |
|  | Beechwood xylan | - |  |
|  | Birchwood xylan | No | 60 |
| TsXynB | Xylobiose | - |  |
|  | Xylotriose | - |  |
|  | pNPX | Yes | 30 |
|  | pNPA | Yes | 30 |
|  | pNPGal | No | 30 |
|  | pNPG | Yes | 30 |
|  | Beechwood xylan | - |  |
|  | Birchwood xylan | - |  |
| JB13GH39 | Xylobiose | Yes | 42 |
|  | Xylotriose | Yes | 42 |
|  | pNPX | 90.3 U/mg ± 8.3 | 42 |
|  | pNPA | No | 42 |
|  | pNPGal | No | 42 |
|  | pNPG | - |  |
|  | Beechwood xylan | No | 42 |
|  | Birchwood xylan | No | 42 |
| Xyl21 | Xylobiose | Yes | 23 |
|  | Xylotriose | Yes | 23 |
|  | pNPX | 17.6 | 23 |
|  | pNPA | - |  |
|  | pNPGal | - |  |
|  | pNPG | - |  |
|  | Beechwood xylan | Yes | 23 |
|  | Birchwood xylan | Yes | 23 |
| Coxyl A | Xylobiose | Yes | 4 |
|  | Xylotriose | Yes | 4 |
|  | pNPX | 3930 U/mg ±132 | 4 |
|  | pNPA | - |  |
|  | pNPGal | - |  |
|  | pNPG | - |  |
|  | Beechwood xylan | Yes | 4 |
|  | Birchwood xylan | - |  |

- Not assayed
